# Learning how to swim in 5- to 12-year-old children: a scoping review of evidence-based motor learning methods

**DOI:** 10.3389/fspor.2025.1505301

**Published:** 2025-02-12

**Authors:** Carola Minkels, John van der Kamp, Ralph de Vries, Peter J. Beek

**Affiliations:** ^1^ InnoSportLab de Tongelreep, Eindhoven, Netherlands; ^2^ Department of Human Movement Sciences, Amsterdam Movement Sciences, Vrije Universiteit Amsterdam, Amsterdam, Netherlands; ^3^ Medical Library, Vrije Universiteit Amsterdam, Amsterdam, Netherlands

**Keywords:** swimming, learning methods, children, skill acquisition, motor learning

## Abstract

**Background:**

Swimming is widely acknowledged for its safety and health benefits. Across the world children are receiving swimming lessons in which a variety of learning methods are employed. However, little is known about the effectiveness of those methods, and a comprehensive overview of pertinent research is lacking. Such an overview is needed for both researchers and instructors seeking to improve swimming skill acquisition in children.

**Objective:**

This scoping review aims to provide an overview of studies examining the effectiveness of motor learning methods for the acquisition of swimming skills by 5- to 12-year-old children, including an evaluation of their theoretical underpinnings, methodological quality, and core findings.

**Methods:**

This scoping review adhered to the PRISMA guidelines and followed Tricco et al.'s framework for conducting and reporting scoping reviews. Five bibliographic databases were systematically searched. Peer-reviewed studies in all languages published before 2025 were considered. Studies focusing on children with water-related fear were included. Gray literature, non-peer-reviewed studies and studies on specific groups (e.g., young, competitive swimmers or children with disabilities), or cognitive/motivational outcomes were excluded. Review selection and characterization were performed by three independent reviewers using pretested forms.

**Results:**

A total of 23 studies were included, which were classified into three main categories: traditional motor learning methods (*n* = 4), contemporary methods (*n* = 1), and atheoretical methods (*n* = 18). Traditional methods focused on video-based instruction and feedback (*n* = 4). Contemporary methods involved a single study on a non-linear swimming program (*n* = 1). Atheoretical methods were further classified into learn-to-swim programs (*n* = 12), learning environments (*n* = 3), and assistive devices (*n* = 3). Most studies (87%) reported a positive effect of the motor learning method under investigation during practice. However, significant methodological limitations were identified. Specifically, 87% of studies did not incorporate retention or transfer tests, 35% lacked control or comparison groups, and 48% did not provide detailed descriptions of the investigated intervention(s). Additionally, 83% of studies were not explicitly grounded in theoretical frameworks, except for the video-based studies and the study on a non-linear swimming program.

**Conclusion:**

The literature on this topic is scarce, generally atheoretical and of questionable methodological quality. Addressing these shortcomings in future research will improve the evidence-base for the effectiveness of theoretically inspired learning methods for the acquisition of swimming skills in children, and their long-term retention and transfer, which in turn might result in evidence-based innovations in swimming lessons.

**Systematic Review Registration:**

PRISMA (RRID:SCR_018721).

## 1 Introduction

Since time immemorial, humans have ventured to move and survive in aquatic environments, presumably driven by both curiosity and necessity. The earliest recordings of swimming date back to 7,000-year-old Stone Age Paintings. In 1538, German language professor Nikolaus Wynmann published what is widely regarded as the first book on swimming, which includes descriptions of survival techniques such as the breaststroke to reduce the risk of drowning (1). Since then, many authors have followed suit. In the same century, Everard Digby wrote “*De Arte Aatandi Libri Duo”*, which contains illustrations of the backstroke, back crawl, front crawl, and breaststroke (2). In the 17th and 18th centuries, pioneers such as Melchisédech Thévenot, Benjamin Franklin, and Johann Christoph Friedrich GutsMuths made significant contributions to the subject of swimming as well (3–5). These books contain many interesting ideas on how to perform, practice, and teach swimming skills. For instance, GutsMuths described various swimming, diving, and jumping techniques and introduced innovative concepts such as the “frog method” for learning breaststroke leg movements and the use of a pentagonal bench for dryland swimming practice (5). These classical works illustrate the long-standing interest in swimming and the acquisition of swimming skills.

The unwavering interest in swimming of humankind is not surprising given the multiple benefits of swimming. Being able to swim significantly reduces the risk of drowning. Swimming is also a highly enjoyable activity that offers extensive health benefits as a full-body workout requiring both endurance and strength [cf (6)]. Moreover, swimming is characterized by a low incidence of injury, making it an ideal lifelong fitness activity suitable for all ages (7). Consequently, beyond the obvious safety considerations, learning to swim can also serve as an introduction to swimming as a recreational activity, be it at a club or otherwise.

To reap the long-term health benefits and enjoyment of swimming, swimming skills need to be maintained once acquired (8, 9). However, swimming skill retention is often suboptimal. For example, in a study with participants of a Dutch national swimming program, van der Weijden-van Rooden (10) reported that only 56% of children could successfully perform 5 basic swimming skills (e.g., exiting the water, entering the water, 25-m backstroke, 25-m breaststroke, 25-s water treading) 18 months after initially acquiring them. While some efforts have been made to investigate the optimization of swimming skill retention in children, research on this important topic with significant societal ramifications remains limited (11–13).

Nowadays, most children learn to swim during formal swimming lessons, during which a broad variety of motor learning methods are applied. A rough distinction may be made between traditional and contemporary motor learning methods. In accordance with Schöllhorn's foundational studies (14–16), we define traditional learning methods as methods that are (implicitly or explicitly) based on the assumption that for each skill (e.g., breathing, floating, diving) and stroke type (e.g., front crawl, breaststroke, backstroke) an ideal movement pattern exists that should be successively approximated through repetitive practice and training, resulting in stable internal representations of the required movement pattern in the form of motor programs or sensory-motor schemas. Instructions are based on this ideal, correct movement pattern and feedback is directed at nullifying the difference (or “error”) between the performed and ideal movement pattern. Another defining feature of traditional learning methods is that learning is viewed as a linear process in the sense that the (sub)actions composing complex skills are learned (and taught) in serial order, starting from the simplest to the more complex, finally culminating in the full-blown execution of the complex skill of interest [e.g., (17)]. This line of thinking has a long history in the study of motor learning and can be found in the classical works of Henry (18), Fitts and Posner (19), and Adams (20), as well as in the practical fields of human movement (e.g., sports, physical education, and dance), as evidenced by the type of instructions and feedback that are still often employed in those fields.

By contemporary learning methods we mean all learning methods that have broken with this traditional perspective, most notably by abandoning the concept of an ideal movement pattern and the corresponding emphasis on movement-oriented instructions and feedback. Such motor learning methods have emerged from around 1990 onwards and include implicit learning (21), learning with an external focus of attention (22), differential learning (14), and the constraints-led approach (23), also dubbed ecological dynamics (24). In a nutshell, according to these methods, the learning of complex perceptual-motor skills benefits from: reducing the amount of explicit knowledge about movement being accumulated during practice (implicit learning), focusing attention on the effect of the movement rather than on the movement itself (external focus of attention), making deliberately large variations in consecutive movements to optimize individual motor solutions (differential learning), and manipulating environmental, organismic and task constraints such that individual perceptual-motor solutions are explored and embraced (ecological dynamics, constraints-led approach). These methods have in common that they view skill learning as an individual, explorative, and non-linear process, in which detailed instructions and feedback about the desired ideal movement patterns during practice are deemed less useful, if not counterproductive.

Both traditional and contemporary motor learning methods have been incorporated into swimming lessons to facilitate the acquisition of swimming skills in children. According to Singh^1^, swimming instructors extensively use verbal techniques such as instruction, explanation, description, feedback, and the use of cue words to support the acquisition of swimming skills in a traditional manner, i.e., with the aim to implement the required movement patterns. In contrast, Invernizzi et al. (25) designed a non-linear swimming program in which children practice aquatic skills in changing circumstances, deliberately created by the program instructors to encourage the exploration of various movement solutions (23, 25–27). In like spirit, Papadimitriou and Loupos (28) introduced a playful training method, which integrates experiences from daily life into exercises and incorporates various objects to support the acquisition of swimming skills in children.

Some contemporary motor learning methods, notably external-focus-of-attention learning (29, 30) and analogy learning (31), a form of implicit learning, have been investigated in adults learning to swim. However, the effects of motor learning methods in adults may not directly translate to children, because they are in a different developmental stage than adults, with different cognitive and physical abilities (32–34). Since most individuals learn swimming skills during early childhood at primary school, it is important to specifically examine if the positive effects of these motor learning methods also hold for children within this age group (35).

The foregoing considerations show that many different learning methods are or can be employed in swimming lessons. But what evidence can be gleaned from the scientific literature regarding the effectiveness of these learning methods in facilitating the practice and long-term learning (i.e., retention and transfer) of swimming skills in children? How strong are the theoretical underpinnings of those methods and how solid is the evidence from a methodological point of view? An initial literature scan revealed that comparatively little research has been conducted on swimming skill acquisition in children and that no comprehensive overview of pertinent research on this topic is currently available. However, such an overview would be useful for swimming researchers and instructors alike. For researchers, it would provide a convenient survey of the research that has been conducted on the topic to date, including the unveiling of research gaps they might wish to fill in future research. For swimming instructors, it would provide a helpful synopsis of learning methods with proven effectiveness that they might consider applying in their lessons. In this contribution, we therefore aimed to provide a comprehensive review of studies examining the effectiveness of motor learning methods for the acquisition of swimming skills by 5- to 12-year-old children, including an evaluation of their theoretical underpinnings, methodological quality, and core findings.

In our initial literature scan, we found two reviews related to the topic of interest, albeit they were not specifically focused on letting children acquire swimming skills through motor learning methods. Leavy et al. (36) reviewed drowning prevention interventions, focusing on the educational and knowledge aspects of drowning prevention, the implementation of water access barriers, and the importance of supervision measures. Mekkaoui et al. (37) performed a systematic review focusing on the effectiveness of teaching methods, rather than motor learning methods, for imparting aquatic skills and knowledge to young children (aged 4-6 years) to enhance water safety. Although teaching and learning are closely intertwined in education, they represent distinct conceptual perspectives, both in terms of practical applications and theoretical underpinnings. Teaching methods are primarily concerned with the external process of transferring knowledge from instructor to learner (38, 39), whereas motor learning methods emphasize the internal processes involved in skill acquisition and retention, whereby learners develop and refine motor abilities through practice (8, 39). The present review focuses specifically on motor learning methods and thus takes a different perspective than Mekkaoui et al. (37), however without ignoring the interrelatedness of teaching and learning. Moreover, while the scoping review by Mekkaoui et al. (37) primarily concentrated on water safety, our focus is specifically on the learning of swimming skills. Finally, Mekkaoui et al.'s (37) review targeted preschoolers aged 4–6 years, whereas many children also learn to swim at older ages during primary school (35). Recognizing these limitations in their research, Mekkaoui et al. (37) made a plea for future research on examining the effectiveness of motor learning methods in learning swimming skills to primary school-aged children. Hence, although useful, the reviews by Leavy et al. (36) and Mekkaoui et al. (37) did not specifically address the effectiveness of motor learning methods that might be invoked in swimming lessons to children at the swimming lesson age. This scoping review aims to fill this gap and can thus be seen as complementing those previous reviews.

## 2 Methods

### 2.1 Protocol and registration

This review was reported in accordance with the Preferred Reporting Items for Systematic Reviews and Meta-Analyses [PRISMA (RRID:SCR_018721)], using Tricco et al.'s (40) framework for conducting and reporting scoping reviews.

### 2.2 Eligibility criteria

Studies were included if they evaluated the effectiveness of motor learning methods or tools for the acquisition of swimming or aquatic skills in children aged between 5 and 12 years. 'Swimming skills' refers to the proficiency in various strokes such as front crawl, back crawl, and breaststroke, while “aquatic skills” refers more broadly to all (motor) skills performed in aquatic environments, i.e., besides the basic swimming strokes, also balance and buoyancy, breathing, underwater orientation, and more advanced strokes like the butterfly (41–43). Aquatic skills were included in the present review because they encompass swimming skills, implying that swimming skills may be addressed in studies focusing on aquatic skills, or are advertised under that rubric. Furthermore, we included studies focusing on teaching methods because these methods may also result in motor learning and may have been studied in this capacity. We excluded gray literature and non-peer-reviewed studies, as the methodological quality of these studies is less reliable. Furthermore, we excluded studies that focused on specific groups, such as performance improvement in young, competitive swimmers or children with disorders or disabilities, because the swimming levels in these groups differ from the general child population receiving swimming lessons. However, studies focusing on children with water-related fear were incorporated, because fear constitutes a common barrier to learning to swim and plays a role in all swimming lessons for children. Lastly, we excluded studies that only measured cognitive or motivational outcomes, as our focus was specifically on the acquisition of motor skills rather than on cognitive or motivational skill enhancement.

### 2.3 Information sources and search

We conducted systematic searches in the bibliographic databases EBSCO/ERIC, EBSCO/SPORTDiscus, EBSCO/APA PsycInfo, Web of Science (Core Collection), and Scopus from the inception of those databases up to December 23, 2024, in collaboration with a librarian (RV) with vast expertise in conducting systematic literature searches. The databases in question were selected because they are comprehensive and cover a broad range of disciplines. The following terms were used (including synonyms and closely related words) as index terms or free-text words: “Motor skill learning”, “Skill development”, “Teaching”, “Aquatic sports”, “Swimming”, and “Children”. The full search strategies for all databases are listed in Supplementary Material I. Only electronic databases were searched. Studies written in all languages were accepted for inclusion, and studies were not excluded based on publication date. Duplicate articles were excluded by a medical information specialist using Endnote X20.0.1 (Clarivatetm), following the Amsterdam Efficient Deduplication (AED) method (44) and the Bramer method (45).

### 2.4 Selection of sources of evidence

Following the search, all identified citations were gathered and imported into Endnote 21 (Clarivate Analytics, PA, USA), where the remaining duplicates were eliminated. Subsequently, the citations were uploaded into Rayyan for screening (46). To enhance the consistency of screening, a pilot test was conducted involving three reviewers (CM, JK, PJB). The reviewers independently screened and discussed a random sample of 50 titles/abstracts adhering to predetermined inclusion criteria. A consensus agreement of 83.8% was achieved during this pilot test, which was deemed adequate to proceed with the screening process. No adjustments were made to the inclusion criteria following the pilot test. Subsequently, the same three reviewers independently screened all potentially relevant titles and abstracts for eligibility against the inclusion criteria of the review. If necessary, the full-text article was checked for eligibility. Full-text articles were obtained through institutional holdings available to the authors. In instances where the full-text was unretrievable through this avenue, attempts were made to contact the source author or journal for assistance in procuring the article. Reasons for the exclusion of articles at the full-text stage were documented and reported.

### 2.5 Data charting process and data items

Data was extracted from all full-text articles included in the scoping review by three independent reviewers, utilizing a data extraction tool developed by the lead author (CM). A tool provided by the Joanna Briggs Institute (JBI) (47) served as the starting point. This tool underwent a review process by the other two reviewers (JK and PJB). The extracted data included specific details such as author(s), year of publication, country where the study was conducted, participants (e.g., mean age, percentage male, sample size), study design, aims, theoretical framework, methodology (e.g., type of motor learning method, to-be-learned skill, measurement instrument, presence of control group, intervention duration), and key findings pertinent to the review questions (see Supplementary Material II).

As regards the measurement instrument used, we assessed whether the to-be-learned swimming skills were measured subjectively or objectively. Subjective measures include expert assessments of perceived progress in aquatic and swimming skills, typically involving rating scales, as well as children's self-assessments, often collected by means of questionnaires administered directly to the children. In contrast, objective measures are derived from kinematic measurements and analyses, which include swimming velocity, stroke length, and stroke count. This distinction was made because objective measurements typically focus on the outcome of the movement. Furthermore, they are generally more accurate, more consistent, and less prone to bias compared to subjective measurements, resulting in a higher reliability and validity of the research findings (48). Conversely, subjective measurements focus on the specific mechanics involved in performing a particular movement. They are more representative of typical swimming lessons, thereby enhancing their practical application (49).

A pilot was performed with the draft data extraction tool by all three reviewers using the first five papers from the included studies in alphabetical order. They tested the form to ensure that all relevant results were covered. Minor adjustments were made to the data extraction tool following the pilot test. Differences in judgment that arose between the reviewers at each stage of the selection and data charting process were resolved through discussion.

### 2.6 Critical appraisal of individual sources of evidence

To assess the overall reliability of the included studies and, consequently, the effectiveness of the motor learning methods examined, we conducted a critical appraisal of the methodologies used in those studies. This process was carried out by three reviewers (CM, JK, and PJB), who extracted relevant data from each study, including the theoretical framework employed, if any, the presence of post-tests, retention tests, transfer tests, control groups, subjective or objective measurement instruments, and the provision of specific program details. This information was incorporated into the data extraction tool (see Supplementary Material II).

### 2.7 Synthesis of results

Both descriptive and evaluative results were extracted and assembled. The studies were classified into three overarching categories based on their theoretical underpinnings: traditional motor learning methods, contemporary motor learning methods, and atheoretical motor learning methods, i.e., motor learning methods lacking a theoretical foundation. Further subdivisions were made in each category to summarize the research in a logical and easily accessible manner (see results). For each subcategory, we provided summaries and frequency counts of the overall characteristics (e.g., year of publication and country), the characteristics of the interventions (e.g., participants, intervention duration, to-be-learned skill), a critical appraisal of the used methodologies, the aims, and the key findings regarding retention and transfer within the respective category.

## 3 Results

### 3.1 Selection of sources of evidence

**Figure 1:**
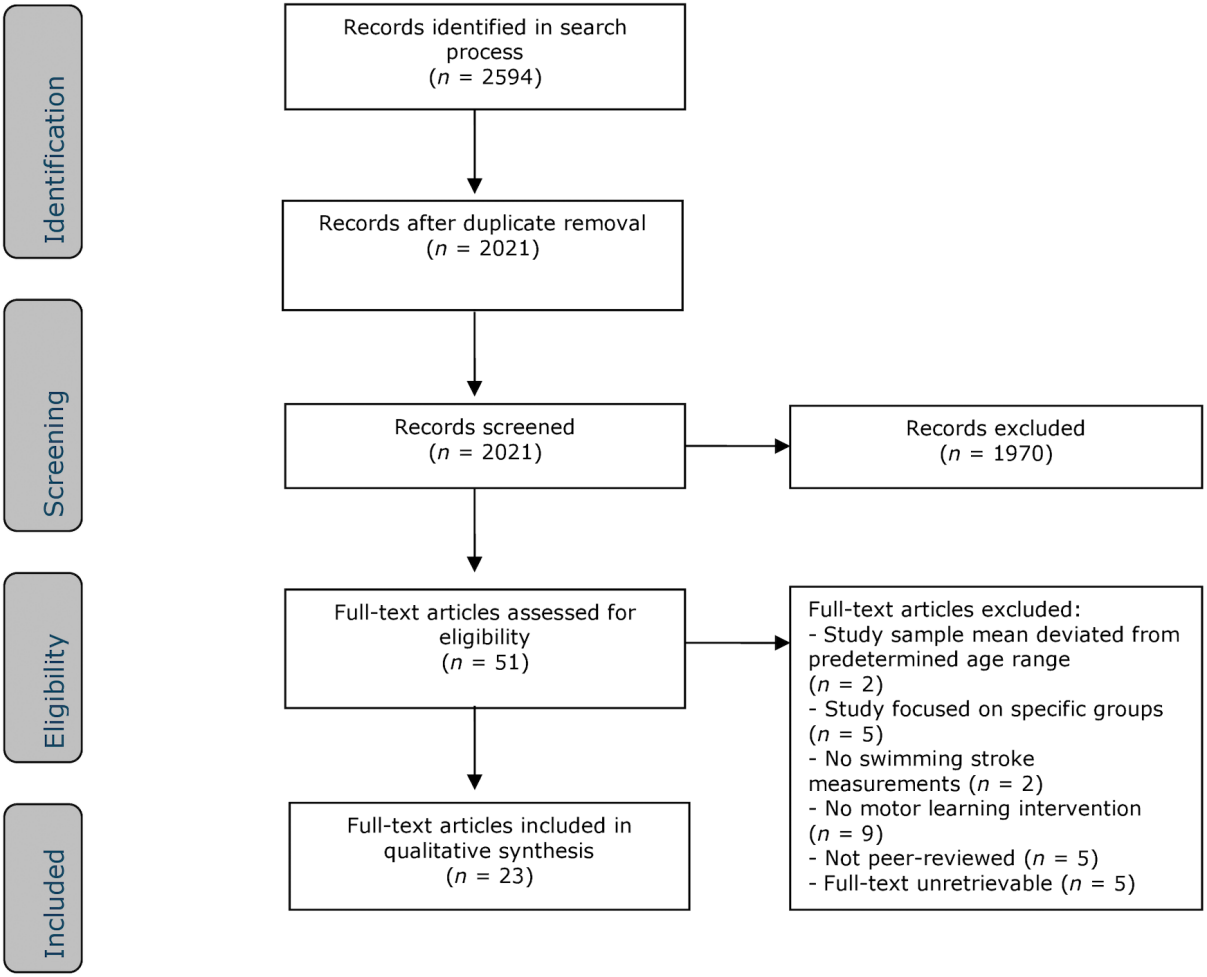
Flowchart of the search, selection, and exclusion procedure.

The literature search yielded a total of 2,594 references: 333 in ERIC, 1090 in SPORTDiscus, 190 in APA PsycInfo, 464 in Web of Science, and 517 in Scopus. After removing duplicates of references across databases, 2,021 references remained. Based on title and abstract screening, 1,970 studies were excluded, leaving 51 full-text articles to be retrieved and assessed for eligibility. From these, 28 articles were excluded for the following reasons: the mean age of the study sample deviated from the predetermined age range (*n* = 2), the study focused on specific groups (*n* = 5), the study did not measure swimming performance (*n* = 2), the study lacked a motor learning intervention (*n* = 9), the study was not peer-reviewed (*n* = 5), and the full text was unretrievable (*n* = 5). The remaining 23 studies were included in this review. The flow chart of the search, selection, and exclusion process is depicted in Figure 1.

We categorized the 23 studies into three primary categories: traditional motor learning methods, contemporary motor learning methods, and atheoretical motor learning methods. Traditional learning methods encompassed the use of video for instruction and feedback (*n* = 4). Contemporary learning methods comprised a single study examining a non-linear swimming program (*n* = 1). Finally, the atheoretical learning methods consisted of learn-to-swim programs (*n* = 12), manipulations of the learning environment (*n* = 3), and the use of assistive devices (*n* = 3). The main characteristics of the studies are detailed in Table 1. Table 2 outlines the aims, adopted theoretical frameworks, and key findings of the studies, while Table 3 provides a critical appraisal of the methodologies used, followed by suggestions for improving their methodological quality.

**Table 1:** Main characteristics of included studies.

Author(s)	Country	Population	Type of intervention	Study design	Presence of comparison group	To-be-learned skill	Measurement instrument	Intervention duration
Traditional: use of video for instruction or feedback								
Bunker et al. (50)	United States	Number *n* = 36 Age 4.5–8.5 years.	Video feedback	Pretest-intervention-posttest	Yes (video + auditory feedback vs. auditory feedback)	Freestyle flutter kick	Perceived progress observed by expert	Number of sessions *n* = 4 Duration of one session *t* = 15 minutes Total duration *t* = 4 weeks
Clark & Ste-Marie (51)	Canada	Number *n* = 33 (*F* = 20, *M* = 13) Age 8.3 (±1.2) years.	Self-as-a-model intervention	Pretest-intervention-retention (24 hours)	Yes (self-modeling vs. video feedback vs. control)	1. Stroke of choice (front crawl, back crawl, breaststroke, elementary backstroke, butterfly) 2. Motivational beliefs	1. Perceived progress observed by expert 2. Questionnaires	Number of sessions *n* = 6 Duration of one session *t* = 30 minutes Total duration *t* = 6 days
Da Silva Pinto Marques-Dahi et al. (52)	Brazil	Number *n* = 20 (*F* = 12, *M* = 8) Age 12 (±0.63) years.	Video + verbal instruction	Pretest-intervention-retention (1 week)-transfer	Yes (video + arm instruction vs. video + interaction arm and breathing instruction vs. video only)	Front crawl	Perceived progress observed by expert + kinematic measurements	Number of sessions *n* = 4 Total duration *t* = 2 weeks
Weiss et al. (53)	United States	Number *n* = 24 (*F* = 6, *M* = 18) Age 6.2 (±0.90) years.	Video instruction	Pretest-intervention-posttest-retention (4 days)	Yes (peer-mastery vs. peer-coping vs. control)	1. Aquatic skills (blowing bubbles, submersion, supported prone float, prone float with kick, front crawl, back float) 2. Self-efficacy 3. Anxiety management	1. Perceived progress observed by expert 2. Questionnaires 3. Questionnaires	Number of sessions *n* = 3 Duration of one session *t* = 20 minutes Total duration *t* = 3 days
Contemporary: non-linear swimming program								
Invernizzi et al. (25)	Italy	Number *n* = 100 (*F* = 53, *M* = 47) Age 5.9 (±0.3) years.	Non-linear pedagogy	Pretest-intervention-posttest	Yes (linear pedagogy vs. non-linear pedagogy)	1. Aquatic skills (41) 2. Perceived aquatic skills	1. Perceived progress observed by expert 2. Questionnaires	Number of sessions *n* = 30 Duration of one session *t* = 50 minutes Total duration *t* = 15 weeks
Atheoretical: learn-to-swim programs								
Bitang et al. (54)	Romania	Number *n* = 16 (*F* = 4, *M* = 12) Age 5–7 years.	Not specified	Pretest-intervention-posttest	No	Aquatic skills (floating, gliding on water surface, breathing, front crawl)	Perceived progress observed by expert	Number of sessions *n* = 36 Total duration *t* = 3 months
Calverley et al. (55)	Australia	Number *n* = 105 (*F* = 44, *M* = 61) Age Group 1: 8.1 years. Group 2: 10.9 years.	Not specified	Pretest-intervention-posttest	No	1. Water safety knowledge 2. Aquatic skills (floating, swimming, safe water entry and exit, rescue skills, survival swimming)	1. Questionnaires 2. Perceived progress observed by expert	Number of sessions *n* = 10 Duration of one session *t* = 60 minutes Total duration *t* = 5 days or 10 weeks
Čižas & Milašius (56)	Lithuania	Number *n* = 25 (*F* = 13, *M* = 12) Age 6.91 (±0.58) years.	Not specified	Pretest-intervention-posttest	No	Aquatic skills (breathing, floating, gliding on water surface, breaststroke, backstroke, jumping into water)	Perceived progress observed by expert + kinematic measurements	Number of sessions *n* = 20 Duration of one session *t* = 45 minutes Total duration *t* = 10 weeks
Frankl (57)	United States	Number *n* = 78 (*F* = 39, *M* = 39) Age 7–10 years.	Not specified	Pretest-intervention-posttest	No	1. Aquatic skills (58) 2. Self-esteem 3. Attitudes toward swimming	1. Perceived progress observed by expert 2. Questionnaires 3. Questionnaires	Number of sessions *n* = 21 Duration of one session *t* = 35 minutes Total duration *t* = 1 year
Jurak et al. (59)	Slovenia	Number *n* = 370 Age 8–9 years.	Content and duration swimming program	Pretest-intervention-posttest	Yes (experimental 1 vs. experimental 2 vs. control)	Breaststroke	Perceived progress observed by expert	Number of sessions *n* = 10 or 15 Duration of one session *t* = 60 or 90 minutes Total duration *t* = 10 weeks
Kotliarov (60)	Russia	Number *n* = 40 (*F* = 20, *M* = 20) Age 7–8 years.	Ratio swimming front- and back crawl	Pretest-intervention-posttest	Yes (60:40 ratio vs. 50:50 ratio)	Front and back crawl	Perceived progress observed by expert + kinematic measurements	Number of sessions *n* = 80 Duration of one session *t* = 45 minutes Total duration *t* = 10 months
Kováčová et al. (61)	Slovakia	Number *n* = 60 Age 8.63 years.	Short intensive swimming course	Pretest-intervention-posttest	No	Aquatic skills [jumping into water, flutter kick with kickboard 25 m, diving, and catching puck, swimming (freestyle or backstroke)]	Perceived progress observed by expert	Number of sessions *n* = 5 Duration of one session *t* = 45 minutes Total duration *t* = 5 days
Mirvić & Rasidagić (62)	Bosnia & Herzegovina	Number *n* = 245 (*F* = 0, *M* = 245) Age 8–10 years.	Not specified	Pretest-intervention-posttest	No	Aquatic skills (safe water entry, submersion, underwater breathing, prone float, back float, gliding on water surface, jumping into water, swimming)	Perceived progress observed by expert	Number of sessions *n* = 12 Duration of one session *t* = 90 minutes Total duration *t* = 12 days
Moncrieff et al. (63)	United States	Number *n* = 54 Age 5–11 years.	Frequency swimming lessons	Pretest-intervention-posttest	Yes (6 sessions in 2 weeks vs. 6 sessions in 3 weeks)	Aquatic skills (submersion, vertical float, face float, back float, swimming)	Perceived progress observed by expert	Number of sessions *n* = 6 Duration of one session *t* = 25 minutes Total duration *t* = 2 or 3 weeks
Moura et al. (64)	Brazil	Number *n* = 31 (*F* = 15, *M* = 16) Age 8.0 (±0.86) years.	Focus of swimming program	Pretest-intervention-posttest	Yes (aquatic skills acquisition vs. swimming skills acquisition)	Aquatic skills (41)	Perceived progress observed by expert	Number of sessions *n* = 12 Total duration *t* = 12 weeks
Sheyko & Pashchenko (65)	Ukraine	Number *n* = 30 Age 6–8 years.	Aquatic games	Intervention- posttest	Yes (use of games vs. no use of games)	Front and back crawl	Perceived progress observed by expert	Number of sessions *n* = 36 Duration of one session *t* = 60 minutes Total duration *t* = 12 weeks
Susnara et al. (66)	United States	Number *n* = 200 (*F* = 88, *M* = 112) Age 4–14 years.	Not specified	Pretest-intervention-posttest	No	1. Water safety knowledge 2. Aquatic skills (67) 3. Value for swimming	1. Questionnaires 2. Perceived progress observed by expert 3. Questionnaires	Number of sessions *n* = 16 Duration of one session *t* = 40–50 minutes Total duration *t* = 4 weeks
Atheoretical: learning environment								
Button et al. (11)	New Zealand	Number *n* = 98 (*F* = 44, *M* = 54) Age 9.0 (±1.3) years.	Open-water swimming	Pretest-intervention-posttest-retention (3 months)	No	1. Water safety knowledge 2. Aquatic skills (safe water entry and exit, floating, submersion, obstacle course, rescue skills, swimming)	1. Questionnaires 2. Perceived progress observed by expert	Number of sessions *n* = 13 Total duration *t* = 3 days
Costa et al. (68)	Portugal	Number *n* = 98 Age 4.4 (±0.5) years.	Water depth	Intervention-posttest (6 months)-retention (12 and 18 months)	Yes (shallow vs. deep water)	Aquatic skills (41)	Perceived progress observed by expert	Number of sessions *n* = 144 Duration of one session *t* = 40 minutes Total duration *t* = 18 months
Rocha et al. (69)	Portugal	Number *n* = 21 Age 4.7 (±0.51) years.	Water depth	Pretest-intervention-posttest	Yes (shallow vs. deep water)	Aquatic skills (41)	Perceived progress observed by expert	Number of sessions *n* = 48 Total duration *t* = 6 months
Atheoretical: use of assistive devices								
Misimi et al. (70)	Slovenia	Number *n* = 40 (*F* = 20, *M* = 20) Age 10.5 (±0.5) years.	Use of goggles and snorkel in children with fear of water	Pretest-intervention-posttest	Yes (goggles and snorkel vs. no goggles and snorkel)	Aquatic skills (41)	Perceived progress observed by expert	Number of sessions *n* = 5 Duration of one session *t* = 45 minutes Total duration *t* = 4 weeks
Misimi et al. (71)	Slovenia	Number *n* = 40 (*F* = 20, *M* = 20) Age 10.5 (±0.5) years.	Use of goggles and snorkel in children without fear of water	Pretest-intervention-posttest	Yes (goggles and snorkel vs. no goggles and snorkel)	Aquatic skills (41)	Perceived progress observed by expert	Number of sessions *n* = 5 Duration of one session *t* = 45 minutes Total duration *t* = 4 weeks
Scurati et al. (72)	Italy	Number *n* = 20 Age 8–9 years.	Use of flotation devices	Pretest-intervention-posttest	Yes (flotation devices vs. no flotation devices)	Front crawl	Perceived progress observed by expert + kinematic measurements	Number of sessions *n* = 10 Duration of one session *t* = 40 minutes

**Table 2:** Aims, theoretical frameworks, and key findings of the included studies.

Author(s)	Aim(s)	Theoretical framework(s)	Key findings
Traditional: use of video for instruction or feedback			
Bunker et al. (50)	To examine the effects of video feedback on the flutter kick swimming skill in two age groups: 4.5–6.5 years and 6.5–8.5 years.	None, but in the discussion, reference is made to Piaget's (73) theory	The effectiveness of acquiring skills with video feedback was superior only for the oldest group (4.5–6.5 years: *F* = 1.5, *p* > 0.05; 6.5–8.5 years: *F* = 4.65, *p* < 0.05)
Clark & Ste-Marie (51)	To examine the impact of two self-as-a-model interventions, namely self-modeling and video feedback, on children's self-regulation of learning and swimming performance.	- Zimmerman's (74, 75) triadic analysis of self-regulatory functioning - Bandura's (76–78) social cognitive theory of observational learning	The self-modeling intervention demonstrated superior swimming performance (*F*(2,30) = 9.38, *p* = 0.001, eta_p_^2^ = 0.44), greater self-satisfaction (*F*(2,30) = 8.91, *p* = 0.001, eta_p_^2^ = 0.44), and higher intrinsic motivation (*F*(2,30) = 14.7, *p* = 0.0001, eta_p_^2^ = 0.49). Although not statistically significant, they also displayed a tendency towards greater self-efficacy beliefs (*F*(2,30) = 3.80, *p* = 0.03, eta_p_^2^ = 0.18) compared to both the video feedback and control interventions. No significant differences were found between the video feedback and control interventions (all *p* > 0.05).
Da Silva Pinto Marques-Dahi et al. (52)	To investigate whether verbal instructions emphasizing the interaction between arm stroke and breathing in the front crawl enhance learning gains when combined with video demonstrations.	None	Enhancing a video demonstration with verbal instruction improves children's learning of the front crawl more effectively than providing the video alone (*F*(2, 17)= 3.72, *p* < 0.05, eta_p_^2^ = 0.30). Furthermore, verbal instructions on the interaction between arm stroke patterns and breathing may lead to even better learning outcomes compared to instructions focusing solely on arm stroke patterns (*F*(2, 17)= 3.72, *p* < 0.05, eta_p_^2^ = 0.30).
Weiss et al. (53)	To examine the effects of peer-coping and peer-mastery models on fearful children's motor performance and psychological responses in the context of swimming.	Bandura's (76–78) social cognitive theory of observational learning	Peer-coping (1) and peer-mastery (2) demonstrated better aquatic skill learning (ES_1_ = −0.28, ES_2_ = −0.44), increased self-efficacy (ES_1_ = −1.22, ES_2_ = −0.50), and reduced fear of swimming (ES_1_ = 1.14, ES_2_ = 1.21) compared to the control intervention. Peer-coping showed superior self-efficacy outcomes compared to peer-mastery (ES = −0.94).
Contemporary: non-linear swimming program			
Invernizzi et al. (25)	To compare the effects of a teacher-centered approach (linear pedagogy) and a student-centered approach (non-linear pedagogy) on motor skill acquisition and children's and parent's perceptions of swimming.	Non-linear pedagogy (26)	Children showed a preference for the non-linear approach, finding it more engaging, whereas the linear program led to greater progress (*p* < 0.05, phi = 0.08) and was deemed more rewarding by parents.
Atheoretical: learn-to-swim programs			
Bitang et al. (54)	To evaluate the effectiveness of swimming means in the acquisition of swimming skills among children aged 5–7 years.	None	Swimming performance improved significantly from the pre-test (*M* = 3.93) to the intermediate test (*M* = 5.07) and further to the post-test (*M* = 6.91).
Calverley et al. (55)	To determine the feasibility and effectiveness of a new child-focused lifesaving, swimming, and water safety program delivered in inland regional areas of Victoria: “Bush Nippers”.	None	The study showed an increase in water safety knowledge among participants under 9 years old (*t*(56) = 3.271, *p* = 0.002, *d* = 0.4), but no change was observed among those under 12 (*p* = 0.091). The assessment of aquatic skills solely relied on post-test results without tracking learning progress.
Čižas & Milašius (56)	To develop a program for the acquisition of swimming skills in children aged 6–7 years and to evaluate its effectiveness.	None	The applied program demonstrated a positive effect on children's acquisition of primary swimming skills.
Frankl (57)	To evaluate the effectiveness of a program that teaches water safety principles, as well as swimming and diving skills, to children from low-income families on swimming and diving skills and self-esteem.	None	Swimming ability showed a significant improvement from the pre-test (*M* = 4.26) to the post-test (*M* = 6.06) (*t*(69) = 15.49, *p* < 0.0001). Self-esteem also increased significantly from the pre-test to the post-test (*t*(74) = 2.11, *p* = 0.038). Additionally, students’ attitudes toward swimming improved significantly from the pre-test to the post-test (*t* = 3.7, *p* = 0.001).
Jurak et al. (59)	To assess the effectiveness of two experimental instruction programs (with identical content but different durations) compared to a standard instruction program (featuring different content) in improving swimming skills.	None (apart from some pedagogical notions)	The experimental swimming instruction programs were superior to the control program (*p* = 0.32 between experimental 1 and control; *p* = 0.007 between experimental 2 and control) without being significantly different from each other (*p* = 0.587).
Kotliarov (60)	To determine the optimal ratio of front crawl to back crawl instruction (e.g., 50/50% vs. 60/40%) for acquiring swimming skills in children aged 7–8 years.	None	The 60/40% ratio of front crawl to back crawl instruction was more effective for acquiring swimming skills than the 50/50% ratio (*t* = 3.2, *p* < 0.05).
Kováčová et al. (61)	To examine the effect of a 5-day-long intensive swimming course on swimming acquisition for pupils from elementary school.	None	The five-day swimming course proved effective, with pupils showing significant improvement across all five basic swimming tests (all *p* < 0.0001).
Mirvić & Rasidagić (62)	To evaluate the impact of a 24-hour program designed to let primary school children learn swimming skills.	None	Swimming performance improved significantly from the pre-test to the post-test (*t*(244) = −37.83, *p* < 0.0001).
Moncrieff et al. (63)	To compare the effectiveness of six practice sessions distributed over three days per week versus two days per week on the acquisition of elementary swimming skills in novice swimmers.	None	No statistically significant differences were observed between the group practicing six sessions distributed over three days per week and the group practicing six sessions over two days per week in achieving the six elementary water skills (*p* > 0.05).
Moura et al. (64)	To compare the effects of two learn-to-swim programs—one emphasizing aquatic skills acquisition and the other focusing on swimming skills acquisition —on aquatic readiness and motor coordination in Brazilian school-aged children.	None	Both swimming programs led to improvements in aquatic readiness and motor coordination, with greater improvements in aquatic competence observed after lessons that focused on aquatic skills (*F* = 24.19, *p* < 0.01, eta_p_^2^ = 0.46).
Sheyko & Pashchenko (65)	To establish the effectiveness of using games in water to let primary school-aged children learn swimming skills.	None	The incorporation of aquatic games during swimming lessons resulted in improved front crawl and back crawl performance compared to lessons that did not utilize aquatic games.
Susnara et al. (66)	To examine the impact of an out-of-school swimming program on children and youth from one underserved community.	Theories based on socialization research	The out-of-school swimming program led to significant enhancements in aquatic skills and water safety knowledge (*F*(1) = 130.71, *p* < 0.0001). Participants also developed a positive perception of swimming due to socialization, particularly influenced by the instructors.
Atheoretical: learning environment			
Button et al. (11)	To determine if the developed swimming program is effective for children to acquire and learn aquatic knowledge and skills in open-water environments.	None	Significant improvements were observed in the number of competent children, typically occurring from pre- to post-test and/or from pre-test to retention (all *p* < 0.017)
Costa et al. (68)	To compare the effectiveness of shallow- versus deep-water swimming lessons for 4–5-year-old children in learning aquatic skills over a period of 6, 12, and 18 months of practice.	None	After 6 months of practice, shallow-water lessons resulted in greater water competence compared to deep-water lessons (*Λ* = 0.131, *Χ*^2^ = 43.778, *p* < 0.001). However, this difference was no longer evident after 12 (*Λ* = 0.395, *Χ*^2^ = 19.945, *p* = 0.277) and 18 months (*Λ* = 0.488, *Χ*^2^ = 17.240, *p* = 0.370) of practice.
Rocha et al. (69)	To determine the effects of shallow- versus deep-water swimming lessons on the acquisition of preschoolers’ aquatic skills after 6 months of practice	None, but constructivism (73) is mentioned	Shallow-water swimming lessons led to greater aquatic competence in preschool children after 6 months of practice (*Λ* = 0.119, *Χ*^2^ = 36.124, *p* < 0.001).
Atheoretical: use of assistive devices			
Misimi et al. (70)	To examine the effects of using goggles and snorkel during swimming lessons on the aquatic skills of young non-swimmers with fear of water	None, but the constraints-led approach is mentioned (23)	The use of goggles and a snorkel appears to be more beneficial in swimming lessons for young swimmers with a fear of water compared to lessons that do not involve using goggles (*p* < 0.05), except for activities like blowing bubbles (*F*(0.83, 0.56) = 4.39, *p* = 0.04).
Misimi et al. (71)	To examine the effects of using goggles and snorkel during swimming lessons on the aquatic skills of young non-swimmers without fear of water	None, but the constraints-led approach is mentioned (23)	The use of goggles and snorkel had no significant effect on most aquatic skills of young non-swimmers without a fear of water (*p* > 0.05).
Scurati et al. (72)	To examine the effectiveness of instructional flotation devices on the acquisition of front crawl swimming skills in Italian children aged 8–9 years.	None	The acquisition of the front crawl is not significantly influenced by the use or non-use of instructional flotation devices (*p* > 0.05).

**Table 3:** Critical appraisal of the methodologies used in the included studies.

Author(s)	Theoretical framework	Presence of pos*t*-test	Presence of retention test	Presence of transfer test	Presence of comparison group	Measurement instrument (subjective or objective)	Provision of specific program details	Suggestions to improve methodological quality
Traditional: use of video for instruction or feedback								
Bunker et al. (50)	None, but in the discussion, reference is made to Piaget's (73) theory	Yes	No	No	Yes (video + auditory feedback vs. auditory feedback)	Subjective	Yes	Incorporate retention test, transfer test, objective measurements, and theoretical framework into the study design
Clark & Ste-Marie (51)	- Zimmerman's (74, 75) triadic analysis of self-regulatory functioning - Bandura's (76–78) social cognitive theory of observational learning	No	Yes (24 hours)	No	Yes (self-modeling vs. video feedback vs. control)	Subjective	Yes	Incorporate post-test, long-term retention test, transfer test, and objective measurements into the study design
Da Silva Pinto Marques-Dahi et al. (52)	None	No	Yes (1 week)	Yes	Yes (video + arm instruction vs. video + interaction arm and breathing instruction vs. video only)	Subjective + objective	Yes	Incorporate post-test, long-term retention test, and theoretical framework into the study design
Weiss et al. (53)	Bandura's (76–78) social cognitive theory of observational learning	Yes	Yes (4 days)	No	Yes (peer-mastery vs. peer-coping vs. control)	Subjective	Yes	Incorporate long-term retention test, transfer test, and objective measurements into the study design
Contemporary: non-linear swimming program								
Invernizzi et al. (25)	Non-linear pedagogy (26)	Yes	No	No	Yes (linear pedagogy vs. non-linear pedagogy)	Subjective	Yes	Incorporate retention test, transfer test, objective measurements, and theoretical framework into the study design
Atheoretical: learn-to-swim programs								
Bitang et al. (54)	None	Yes	No	No	No	Subjective	No	Incorporate retention test, transfer test, control group, objective measurements, specific program details, and theoretical framework into the study design
Calverley et al. (55)	None	Yes	No	No	No	Subjective	No	Incorporate retention test, transfer test, control group, objective measurements, specific program details, and theoretical framework into the study design
Čižas & Milašius (56)	None	Yes	No	No	No	Subjective + objective	No	Incorporate retention test, transfer test, control group, specific program details, and theoretical framework into the study design
Frankl (57)	None	Yes	No	No	No	Subjective	No	Incorporate retention test, transfer test, control group, objective measurements, specific program details, and theoretical framework into the study design
Jurak et al. (59)	None (apart from some pedagogical notions)	Yes	No	No	Yes (experimental 1 vs. experimental 2 vs. control)	Subjective	No	Incorporate retention test, transfer test, objective measurements, specific program details, and theoretical framework into the study design
Kotliarov (60)	None	Yes	No	No	Yes (60:40 ratio vs. 50:50 ratio)	Subjective + objective	No	Incorporate retention test, transfer test, specific program details, and theoretical framework into the study design
Kováčová et al. (61)	None	Yes	No	No	No	Subjective	No	Incorporate retention test, transfer test, control group, objective measurements, specific program details, and theoretical framework into the study design
Mirvić & Rasidagić (62)	None	Yes	No	No	No	Subjective	Yes	Incorporate retention test, transfer test, control group, objective measurements, and theoretical framework into the study design
Moncrieff et al. (63)	None	Yes	No	No	Yes (6 sessions in 2 weeks vs. 6 sessions in 3 weeks)	Subjective	No	Incorporate retention test, transfer test, objective measurements, specific program details, and theoretical framework into the study design
Moura et al. (64)	None	Yes	No	No	Yes (aquatic skills acquisition vs. swimming skills acquisition)	Subjective	No	Incorporate retention test, transfer test, objective measurements, specific program details, and theoretical framework into the study design
Sheyko & Pashchenko (65)	None	Yes	No	No	Yes (use of games vs. no use of games)	Subjective	No	Incorporate retention test, transfer test, objective measurements, specific program details, and theoretical framework into the study design
Susnara et al. (66)	Theories based on socialization research	Yes	No	No	No	Subjective	No	Incorporate retention test, transfer test, control group, objective measurements, and specific program details into the study design
Atheoretical: learning environment								
Button et al. (11)	None	Yes	Yes (3 months)	No	No	Subjective	Yes	Incorporate transfer test, control group, objective measurements, and theoretical framework into the study design
Costa et al. (68)	None	Yes	Yes (12 and 18 months)	No	Yes (shallow vs. deep water)	Subjective	Yes	Incorporate transfer test, objective measurements, and theoretical framework into the study design
Rocha et al. (69)	None, but constructivism (73) is mentioned	Yes	No	No	Yes (shallow vs. deep water)	Subjective	Yes	Incorporate retention test, transfer test, objective measurements, and theoretical framework into the study design
Atheoretical: use of assistive devices								
Misimi et al. (70)	None, but the constraints-led approach is mentioned (23)	Yes	No	No	Yes (goggles and snorkel vs. no goggles and snorkel)	Subjective	Yes	Incorporate retention test, transfer test, objective measurements, and theoretical framework into the study design
Misimi et al. (71)	None, but the constraints-led approach is mentioned (23)	Yes	No	No	Yes (goggles and snorkel vs. no goggles and snorkel)	Subjective	Yes	Incorporate retention test, transfer test, objective measurements, and theoretical framework into the study design
Scurati et al. (72)	None	Yes	No	No	Yes (flotation devices vs. no flotation devices)	Subjective + objective	Yes	Incorporate retention test, transfer test, and theoretical framework into the study design

### 3.2 Traditional motor learning methods

#### 3.2.1 Use of video for instruction or feedback

##### 3.2.1.1 Characteristics of sources of evidence

The sample sizes in those studies ranged from 20 to 36 participants. The number of sessions varied from 3 to 6, with each session lasting between 15 and 30 minutes, resulting in total intervention durations ranging from 3 days to 4 weeks. Three studies concentrated exclusively on the acquisition of one specific swimming stroke [e.g., front crawl, freestyle flutter kick, backstroke, etc. (51–53)], while the fourth study focused on the acquisition of a broader set of aquatic skills (54). Two studies also measured children's motivational beliefs (51, 53), acknowledging the significant influence motivational beliefs can exert on the (further) learning process (74–78).

##### 3.2.1.2 Critical appraisal within sources of evidence

A strength of the video-based studies was that most studies (3 out of 4) tested specific hypotheses grounded in theoretical frameworks. These frameworks included Zimmerman's (74, 75) triadic analysis of self-regulatory functioning, Bandura's (76–78) social cognitive theory of observational learning, and Piaget's (73) theory of cognitive development, demonstrating alignment with traditional motor learning methods. Additionally, most video-based studies (3 out of 4) included control groups without video-based instructions or feedback. This indicated that the observed changes or outcomes were truly attributable to the implemented intervention. Furthermore, all studies provided specific details regarding the intervention program, facilitating their practical application directly to swimming lessons.

Despite these strengths, three methodological limitations came to light. First, while most studies (3 out of 4) included retention tests, all retention tests were conducted only one week after practice completion. This limited timeframe makes it impossible to draw conclusions regarding the long-term effects of using video for instruction or feedback. Moreover, it remains unclear whether children preserved their acquired swimming skills over time. Second, only one study used a transfer test (52). Consequently, no conclusions can be drawn regarding the transfer of acquired skills from pool settings to other aquatic environments, such as open water. Hence, it is unknown whether children could apply their acquired swimming skills in different and potentially more hazardous swimming environments. Third, all studies evaluated swimming skills subjectively, relying on the progress as perceived by an expert. Only one study incorporated additional objective measurements of swimming skill acquisition through kinematic analysis (52).

##### 3.2.1.3 Results of individual sources of evidence and synthesis of results

The video-based studies included different interventions with distinct research objectives. One study examined the effect of video feedback on the flutter kick in two age groups: 4.5–6.5 years and 6.5–8.5 years and found that the acquisition of the freestyle flutter kick with video feedback was effective only in the older age group (50).

Weiss et al. (53) studied the effects of peer-coping and peer-mastery models on fearful children's (mean age = 6.2 ± 0.90 years) swimming performance and motivational responses during swimming. Peer-coping models involve individuals of similar age or status demonstrating effective coping strategies, while peer-mastery models involve individuals of similar age or status showing high competence in a specific skill (79, 80). The study revealed that both peer-coping and peer-mastery models promoted aquatic skill acquisition, self-efficacy, and reduced fear compared to practicing aquatic skills without video demonstration. Notably, the peer-coping intervention exhibited higher self-efficacy beliefs than the peer-mastery intervention (53).

Clark and Ste-Marie (51) examined the impact of two self-as-a-model interventions, video feedback and self-modeling, on swimming performance and motivational outcomes in 6- to 10-year-old children. Video feedback implies watching oneself perform the to-be-learned task at the current skill level, whereas self-modeling entails viewing oneself perform the task at a higher skill level achieved through video editing (79–81). The study revealed that the self-modeling intervention resulted in superior swimming performance, greater self-satisfaction, higher intrinsic motivation, and stronger self-efficacy beliefs compared to the video feedback intervention and the intervention that practiced without video demonstration. The video feedback intervention performed better than the control group, but less effectively than the self-modeling intervention (51).

Lastly, a study by Da Silva Pinto Marques-Dahi et al. (52) investigated whether additional verbal instructions emphasizing the interaction between arm stroke pattern and breathing in the front crawl enhanced learning from video demonstrations in children (mean age = 12 ± 0.63 years). The study found that this combined approach improved children's acquisition of the front crawl more effectively than providing a video demonstration alone (52).

Altogether, the literature on video instruction indicates that peer models facilitate swimming skill acquisition, while also boosting motivational beliefs and reducing fear. The effect appears to be indirect: children may identify with peer models, which fosters greater feelings of relatedness. This, in turn, can boost motivational beliefs, reduce fear, and ultimately improve learning outcomes (82). Moreover, combining video instruction with verbal instructions seems to enhance the effectiveness of acquiring swimming skills. This may be due to the improved clarity and the increased personal relevance of the instruction, which likely enhances comprehension and reduces the misinterpretation of the provided information (52).

With respect to video feedback, findings on children's swimming skill acquisition are conflicting, which may be attributed to the varying age ranges across the included studies. It seems that video feedback is effective for children older than 6 years, but less so for younger children. Children aged 6 and older may possess more advanced cognitive, emotional, and social skills, which better equip them to benefit from video feedback. In contrast, younger children may require simpler, more direct, and more concrete forms of guidance (32–34). Self-modeling also demonstrates a positive impact on both skill acquisition and motivational beliefs (51). Like peer modeling, its effectiveness seems to be mediated through improved motivational beliefs (82).

A closer examination of the age ranges studied across video-based interventions—not just those focusing on video feedback— further supports the conclusion that video instruction and feedback are particularly effective for children aged 6 and older. The majority of these motor learning studies were underpinned by traditional motor learning frameworks and included control groups, lending credibility to their findings. However, the long-term effects of video-based studies remain uncertain, as does the transfer of acquired swimming skills to other aquatic environments, due to the lack of research on these aspects.

### 3.3 Contemporary motor learning methods

#### 3.3.1 Non-linear swimming program

##### 3.3.1.1 Characteristics of sources of evidence

One study examined the effectiveness of a non-linear swimming program (25). This study involved a sample of 100 participants and consisted of 30 swimming lessons, each lasting 50 minutes, distributed over 15 weeks. During these lessons, children practiced various aquatic skills.

##### 3.3.1.2 Critical appraisal within sources of evidence

A strength of the investigated non-linear swimming program was that it was explicitly grounded in a specific theoretical framework, known as non-linear pedagogy (26). This framework closely aligns with the constraints-led approach, a contemporary motor learning method (23, 26, 27). Additional strengths of the study were the inclusion of a comparison group (i.e., linear pedagogy) and the provision of specific program details. However, the study also suffered from methodological shortcomings, notably the absence of retention and/or transfer tests and the exclusive reliance on subjective assessments of aquatic skills.

##### 3.3.1.3 Results of individual sources of evidence and synthesis of results

The study compared the effects of a linear swimming program with a non-linear program. In the linear swimming program, children practiced skills in uniform settings and received feedback to refine movements and identify and correct errors, in accordance with traditional motor learning methods. In contrast, in the non-linear swimming program, children practiced aquatic skills using contemporary motor learning methods. This approach entailed practicing in different, continuously changing practice settings without a preconceived linear order. Instructors manipulated individual, task, and environmental conditions to encourage children to explore diverse movement solutions and adapt to the changing conditions. The study's main result was that the children appreciated the non-linear program better, whereas the linear program resulted in better acquisition of aquatic skills and was deemed more appropriate and rewarding by the parents. However, since the study did not incorporate retention and/or transfer tests, it remains unclear which program — linear or non-linear — resulted in better long-term aquatic skill acquisition and transfer to other aquatic environments.

### 3.4 Atheoretical motor learning methods

#### 3.4.1 Learn-to-swim programs

##### 3.4.1.1 Characteristics of sources of evidence

Twelve studies were found on learn-to-swim programs aimed at the enhancement of water safety for children rather than swimming skills/strokes *per se*. The sample sizes across these studies varied widely, ranging from 16 to 370 participants, with a combined total sample of 1,254 participants. The duration of the learn-to-swim programs ranged from 5 to 80 sessions, with sessions lasting between 25 and 90 minutes. The total duration of the interventions ranged from 5 days to 1 year.

In most studies (9 out of 12), children acquired a broad palette of aquatic skills including swimming skills. In two studies children acquired the front and back crawl (60, 65), while in another study the children acquired the breaststroke (59). Two studies additionally focused on teaching water safety knowledge (55, 66), while another couple of studies aimed to enhance children's self-esteem and/or valuation of water activities (57, 66).

##### 3.4.1.2 Critical appraisal within sources of evidence

Significant methodological shortcomings were identified in the learn-to-swim programs. First of all, none of the studies were explicitly grounded in theories of motor learning. Second, all studies employed a pretest-intervention-posttest design to assess the acquisition of the to-be-learned skills, but none of the studies included a retention or transfer test. Third, most studies (7 out of 12) did not include a control or comparison group. Fourth, all studies assessed the to-be-learned swimming skills subjectively by evaluating the perceived progress observed by an expert with rating scales. Only two studies also incorporated objective measurements in the form of kinematic measurements and analyses (56, 60). Lastly, most learn-to-swim programs were described quite generally, with studies often lacking detailed information regarding the specific motor learning methods used.

##### 3.4.1.3 Results of individual sources of evidence and synthesis of results

The learn-to-swim programs encompassed various interventions with different aims. Two studies specifically examined the impact of swimming program content on swimming skill acquisition. One study compared the impact of swimming lessons that incorporated aquatic games with those that did not in the development of children's front crawl and back crawl skills. The findings indicated that lessons incorporating aquatic games were more effective in improving these skills than lessons without those games (65). The second study examined an experimental program that incorporated several elements, including differentiation between children, individualized attention to each child, games, obstacle courses, relays, additional equipment, and the use of music. This experimental approach was compared with a control program without these elements. The study concluded that the experimental program incorporating these elements resulted in superior acquisition of the breaststroke compared to the control program (59).

Two studies examined approaches to swimming skill acquisition. One study compared the effectiveness of emphasizing aquatic skill acquisition vs. swimming skill acquisition. The aquatic skill approach focused on skills critical for water safety, while the swimming skill approach prioritized swimming skills and skills essential for swimming strokes, such as breath control and horizontal buoyancy. The results favored the emphasis on aquatic skills as the more effective approach for developing swimming skills (64). The second study compared two different ratios of front crawl to back crawl instruction (e.g., 50/50% vs. 60/40%) and concluded that the 60/40% ratio was more effective for acquiring swimming skills (60).

Three studies explored the effects of swimming program duration and frequency on the acquisition of aquatic skills (59, 61, 63). Remarkably, one study found no significant effect of swimming program duration (i.e., 15 sessions of 90 minutes vs. 10 sessions of 60 minutes) on the acquisition of the breaststroke (59). Another study found no significant effect of program frequency (6 lessons in 2 weeks vs. 6 lessons in 3 weeks) on aquatic skill acquisition (63). The last study demonstrated that a five-day intensive swimming course (i.e., 5 sessions of 45 minutes) effectively improved five basic aquatic skills (61).

Lastly, six studies did not provide specific details about the investigated interventions. However, all studies reported that their learn-to-swim programs significantly improved children's aquatic skills (54–57, 62, 66).

Altogether, except for two studies regarding program duration and frequency (59, 63), nearly all studies reported a positive effect of the investigated learn-to-swim programs on the acquisition of the to-be-learned skills. Collectively, these studies indicate that the variants intentionally incorporated into learn-to-swim programs are generally effective. However, major methodological shortcomings were evident, including the lack of clear theoretical underpinnings, control groups, objective measurements, and detailed program descriptions. Furthermore, the lack of retention and transfer tests raises questions about the longevity of the observed effects and their generalizability to other aquatic environments.

#### 3.4.2 Learning environment

##### 3.4.2.1 Characteristics of sources of evidence

The sample sizes of these studies varied between 21 participants (69) and 98 participants (11, 68). Also, the duration of the studies varied widely. One study consisted of 13 sessions spread over 3 days (11), another study involved 48 sessions conducted over 6 months (69), and a third study spanned 144 sessions conducted over 18 months (68). In all three studies, children acquired aquatic skills.

##### 3.4.2.2 Critical appraisal within sources of evidence

A strength of the learning environment studies was that two of the three studies incorporated long-term retention tests (at 3, 12, and 18 months, respectively) (11, 68). As a result, it could be established whether the effects of swimming lessons in shallow water and open water persisted over time. Further strengths encompassed the incorporation of comparison groups in the water-depth studies and detailed descriptions of the interventions.

Nevertheless, the studies also demonstrated considerable methodological limitations. First, none of the studies were explicitly grounded in a theoretical framework. No *a priori* hypothesis or ex posteriori explanation was given as to why shallow, deep, and/or open-water swimming lessons might be beneficial for learning. Second, none of the studies incorporated a transfer test. Third, the open-water swimming study lacked a control or comparison group (11), disallowing any conclusion about whether open-water lessons resulted in better learning outcomes than lessons in pools. Lastly, all three studies measured the aquatic skills subjectively.

##### 3.4.2.3 Results of individual sources of evidence and synthesis of the results

Two studies compared the effectiveness of shallow- vs. deep-water lessons for children in acquiring aquatic skills (68, 69). Rocha et al. (69) reported that shallow-water lessons resulted in greater aquatic skill acquisition in children after 6 months of practice compared to deep-water swimming lessons. These findings were supported by Costa et al. (68). However, due to the inclusion of retention tests, Costa et al. (68) also found that this difference was no longer evident after 12 and 18 months of practice. This suggests that shallow and deep water impose different constraints that influence short-term learning (23). Shallow water allows learners to focus on basic skills without the complexities of buoyancy, balance, and fear of deep water, leading to quicker skill acquisition. However, after 12–18 months of practice, learners have adapted to both environments, ultimately leading to similar long-term skill outcomes. The third study examined whether acquiring aquatic skills and water safety knowledge to children in open-water environments is effective and confirmed this to be the case for both short-term and long-term learning (11). Caution is warranted when interpreting the results of the learning environment studies, as the critical appraisal revealed several methodological limitations in all three studies. For example, the absence of transfer tests makes it impossible to determine whether children retained their aquatic skills in other aquatic environments.

#### 3.4.3 Use of assistive devices

##### 3.4.3.1 Characteristics of sources of evidence

Of the three studies examining the use of assistive devices, two focused on goggles and snorkels. These studies were published by the same authors, with the second study building on the first (70, 71). Both studies had a sample size of 40 participants and included five 45-minute sessions conducted over four weeks, during which children acquired aquatic skills. The third study, focusing on flotation devices, involved 20 participants and consisted of 10 sessions lasting 40 minutes each, during which children acquired the front crawl (72).

##### 3.4.3.2 Critical appraisal within sources of evidence

The strengths of the three studies on assistive devices included the use of comparison groups, the application of objective measurements in the study on flotation devices, and the provision of comprehensive details about the intervention programs. Methodological limitations included the lack of explicit theoretical underpinnings, even though the studies on goggles and snorkels made loose reference to the constraints-led approach (23), the omission of retention and transfer tests, and the reliance on subjective assessments of aquatic skills in the studies on goggles and snorkels.

##### 3.4.3.3 Results of individual sources of evidence and synthesis of results

Two studies examined the effects of using goggles and snorkels on aquatic skill acquisition in children. One study focused on children with a fear of water, and the other one on children without fear of water. Incorporating goggles and snorkels during practice was found beneficial for children with a fear of water, except when blowing bubbles. However, their use did not have a significant effect on the acquisition of aquatic skills in children without fear of water. This suggests that the benefits of these devices result from an interaction between individual and task characteristics and are more pronounced in children who experience water fear or anxiety (23). The third study examined the effectiveness of instructional flotation devices on the acquisition of the front crawl in children aged 8-9 years and found that the use or non-use of these devices did not significantly influence learning outcomes. Again, due to the presence of several methodological shortcomings, the results should be interpreted with caution. For example, due to the absence of retention and transfer tests, no conclusions can be drawn regarding the long-term effects or the transfer of skills to other aquatic environments when using goggles, snorkels, or flotation devices.

## 4 Discussion

In this scoping review, we provided an encompassing overview of studies investigating the effectiveness of motor learning methods in the acquisition of swimming skills by 5- to 12-year-old children, including an evaluation of their theoretical underpinnings, methodological quality, and core findings. We selected studies on this topic that have been published to date and assessed the aforementioned properties. Below, we summarize the findings, identify research gaps, suggest promising, if not required, directions for future research, and discuss the relevance of the findings for swimming lessons and instructors. Finally, we discuss the strengths and limitations of the present review itself.

### 4.1 Summary of reviewed studies

This scoping review identified only 23 studies that addressed the effectiveness of motor learning methods in the acquisition of swimming skills by 5- to 12-year-old children. Of these, four studies examined the effectiveness of traditional motor learning methods (i.e., the use of video for instruction and feedback), one study focused on contemporary motor learning methods (i.e., non-linear swimming program), and 18 studies investigated other topics (i.e., learn-to-swim programs, learning environments, or assistive devices) without any explicit theoretical motivation or foundation. This indicates that the research on the topic of interest is largely atheoretical, the prominent developments that have taken place in the scientific field of motor learning notwithstanding. Traditional motor learning theories have evolved further and new, contemporary theoretical approaches to motor learning have emerged since 1990, both of which have great potential for improving swimming education in children. Thus far, however, the potential merits of the science of motor learning for the practice of swimming lessons have remained largely untapped. Traditional and contemporary learning methods have not been sufficiently investigated in relation to swimming skill acquisition in children to draw any firm definitive conclusions regarding their effectiveness in promoting swimming skill acquisition, long-term retention, and transfer to other aquatic environments. Consequently, also no conclusions can be drawn regarding their relative superiority in these regards.

Furthermore, the overall reliability of the reported findings was found to be limited due to the generally poor methodological quality of the studies. Most studies lacked critical methodological elements such as control groups, objective measurements of acquired skills, and detailed descriptions of the swimming programs implemented. Additionally, only two studies included a retention test conducted a substantial period after the completion of practice, specifically, studies on open-water and water-depth learning (11, 68). And only one study had a transfer test, namely a study on video instruction supplemented by verbal cues (52). Consequently, no general conclusions can be made regarding the long-term effectiveness of the motor learning methods investigated or the transfer of the acquired skills to other aquatic environments.

We may thus conclude that the current state of research on learning children swimming skills has largely overlooked insights from the literature on theoretically grounded motor learning methods, except for studies on video-based instruction and feedback and a non-linear swimming program. Furthermore, the methodological quality of the examined studies was generally inadequate. Consequently, the effectiveness of motor learning methods to promote children's acquisition of swimming skills remains largely unknown and inconclusive. This conclusion is akin to that of Leavy et al. (36) and Mekkaoui et al. (37), who observed that most drowning prevention interventions lacked theoretical foundations and underscored that the question of “how to teach swimming skills” requires further investigation.

### 4.2 Directions for future research

To enhance the acquisition of swimming skills by children in swimming lessons, there is a definite need to examine the (relative) effectiveness of both traditional and contemporary motor learning methods, with the goal to make swimming lessons more evidence based. As concluded, neither type of approach has been investigated to a sufficient degree in the context of swimming and swimming lessons to make any strong claim about their effectiveness, let alone relative superiority. Given this situation, the best way forward seems to conduct robust and carefully designed experimental studies on the effectiveness of both types of motor learning methods for mastering swimming skills by children, ideally including critical comparisons between experimental arms on key concepts and theoretically motivated hypotheses. In this manner, useful insights have been obtained in the acquisition of other complex motor skills than swimming, also in children, showing in general that the contemporary learning methods are not inferior to traditional learning methods and in some studies even have the edge (83–85). To start making swimming lessons more evidence based, research is required on key concepts and hypotheses from both types of motor learning methods. In principle, this research can take on many forms, but from our perspective and knowledge of swimming lessons, the following research lines would be valuable to pursue.

To start at the traditional end of the spectrum, it would still be valuable from a practical point of view to examine the effectiveness of the variability of practice hypothesis and corresponding method (86). This method asserts that systematically varying relevant parameters of the motor scheme, which serves a class of movements, optimizes the development of the motor schemes, and thus learning outcomes (86). Building on this premise, future research could investigate the impact of systematically varying factors such as stroke length or stroke rate during practice, comparing it with continuous practice at a constant, correct stroke length and rate in the context of acquiring swimming strokes.

Additionally, the effectiveness of contextual interference could also be explored in swimming lessons (87). Contextual interference pertains to the order in which different motor skills are practiced. It posits that the interference caused by randomly or serially switching between skills, rather than practicing them in separate blocks, forces the individual to engage in deeper cognitive processing and/or provides desirable difficulty levels (88, 89). This, in turn, leads to the development of stronger, more distinct motor schemes and better learning outcomes (87, 90). Building on this concept, future research could investigate the effectiveness of acquiring different swimming strokes in a randomized order vs. practicing each stroke in discrete blocks.

In both types of study, it would be very informative to include a differential learning group with a higher degree of variation, and without preconceived movement invariants that should be accommodated. In this way, the effectiveness of traditional and contemporary motor learning methods of inducing variation during swimming education could be compared, which is an issue of great practical relevance.

Furthermore, when considering contemporary learning methods, we see merit in exploring the impact of implicit learning, which aims to enhance motor learning by minimizing the amount of explicit knowledge a learner accumulates about the skill's desired movement pattern (21, 91). This approach contrasts with the explicit learning methods commonly seen in swimming lessons, where detailed descriptions of movement patterns are provided (18, 20). For example, exploring the use of analogies on the acquisition of swimming skills in children could be valuable, building on the positive effects observed in adults (31). Consequently, future studies might examine the effectiveness of using the “frog-plane-pencil” analogy for acquiring the breaststroke, an analogy that is frequently used by Dutch swimming instructors. Such a study would also allow testing whether the insight that implicitly learned motor skills are less susceptible to deterioration under psychological pressure than explicitly learned motor skills (21, 92) also holds in children. This may be advantageous for children who end up in unfamiliar and dangerous water environments, such as rapids, where they need to perform self-rescue maneuvers.

Likewise, it would also be valuable to examine whether learning with an external focus of attention facilitates the acquisition of swimming skills in children, since Freudenheim et al. (29) demonstrated this to be the case in adults. Future research could, for example, compare the effectiveness of providing children with external focus instructions, such as “push the water back”, as opposed to internal instructions, such as “pull your hands back”, as was done by Stoate and Wulf (30) in trained teenaged swimmers.

We also see merit in further investigating the value of the constraints-led approach for acquiring swimming skills in children. In this approach, learning environments are designed that invite children to explore new skills by adding constraints that are aimed to steer individuals away from or towards particular movement solutions (23). By adopting this approach, rather than prescribing specific movement patterns, the instructor creates different conditions that encourage the child to explore and discover their own adaptive movement pattern (23). Promoting the development of adaptive movement patterns may facilitate the transfer of learned swimming skills to other, more hazardous aquatic environments, such as rivers, lakes, seas, and oceans, thereby potentially enhancing children's safety and adaptability in different water settings.

The study by Invernizzi et al. (25) touched on this approach by comparing a non-linear swimming program with a linear one. However, this study did not include retention or transfer tests, leaving it uncertain whether a non-linear swimming program leads to better long-term swimming skill acquisition or transfer to other aquatic environments than a linear swimming program. Future studies should address this gap.

Thus, although some studies in this scoping review touched on this approach, including also the studies examining the use of goggles and snorkels (70, 71), the potential benefits of the constraints-led approach to acquiring swimming skills remain untapped. This is also recognized by van Duijn et al. (12). Sheaff's (93) recent book on the constraint-led approach to swim coaching contains some suggestions on how this approach might be applied to children, for instance, to have them swim with a weight belt or to change the surface area of their hands. Future research should examine the effectiveness of such suggestions, as well as the effectiveness of using assistive devices for floating (e.g., boards) or propulsion (e.g., flippers) from a learning perspective, in line with the research of van Duijn et al. (12).

In addition to exploring motor learning methods that have not yet been investigated in the context of children acquiring swimming skills, we also recommend further examination of the effectiveness of technical innovations, particularly video technology stratified by age groups. Although studies in this scoping review have demonstrated the potential effectiveness of video instruction, the most effective methods for its use, as well as its practical implementation within swimming lessons, have yet to be determined. For example, future research could examine the impact of different video instructional models on the acquisition of swimming skills in children during swimming lessons.

Lastly, it is important to focus not only on the assessment of learning outcomes but also on the measurements of cognitive and motivational variables. Currently, only 4 out of 23 studies examined this aspect (51, 53, 57, 66). Understanding how to effectively foster intrinsic motivation in children learning to swim is crucial, as higher intrinsic motivation can lead to faster skill acquisition and continued engagement in swimming beyond swimming lessons (82, 94). For instance, using the “frog-plane-pencil” analogy may not only enhance learning but also foster feelings of relatedness and boost intrinsic motivation when children learn the breaststroke compared to more traditional, prescriptive approaches (94). Moreover, as parents also play a significant role in shaping children's motivation for and affinity with swimming, future research should investigate the role of parents in their children's swimming education (25, 82, 95, 96).

### 4.3 Relevance for swimming lessons and instructors

The extant literature on the topic of interest indicates that the use of video for instruction, and potentially feedback, enhances the acquisition of swimming skills in children, particularly when they are 6 years or older. Nevertheless, the integration of video technology in swimming lessons remains limited (97), likely due to the costs and the difficulties involved in making video footage that is readily available for use in swimming pools. However, based on the promising research results, it may be recommended that swimming instructors incorporate video instruction into their lessons to facilitate the acquisition of swimming skills.

### 4.4 Strengths and limitations

In this scoping review, we applied rigorous methods for searching and selecting relevant publications and reporting, as described in the PRISMA guidelines and by Tricco et al.'s (40) framework. In our striving for completeness, the search strategy included five electronic bibliographic databases. The screening and data extraction tool used was pretested by all three reviewers and refined as necessary before implementation. Each citation and article underwent review by three independent reviewers who met regularly to resolve any emerging disagreement. The use of a bibliographic manager (Endnote 21) in conjunction with systematic review software (Rayyan) allowed that all publications were accurately accounted for throughout the process. These rigorous methods ensured the integrity and reliability of the scoping review.

However, despite the adopted rigorous approach, one limitation is worth mentioning. We may not have identified all relevant articles in published literature. For example, we did not screen the reference lists of the sixteen included articles, and we did not contact researchers or experts for additional articles we may have overlooked.

## 5 Conclusion

In this scoping review, we evaluated the current state of research on the effectiveness of motor learning methods in the acquisition of swimming skills by 5-to-12-year-old children. The literature on this topic is scarce and generally of poor methodological quality. Moreover, little efforts have been made to examine the effectiveness of theoretically grounded motor learning methods, which have been proven effective in adults and in learning other complex motor skills in children. It is important to address these gaps, particularly given the need for the application of evidence-based motor learning methods in swimming lessons. Ideally, these methods do not only result in a high transfer and retention of the acquired swimming skills, but also foster a lifelong engagement with swimming among children.

## Data Availability

The original contributions presented in the study are included in the article/Supplementary Material, further inquiries can be directed to the corresponding author.

## Footnotes

^1^Singh M. A generation and classification of the possible learning styles used in the acquisition of basic swimming skills (2004). Unpublished MSc thesis. University of Alberta, Edmonton, Alberta, Canada.
