# Supplementary material for: Learning how to swim in 5- to 12-year-old children: a scoping review of evidence-based motor learning methods
Source: Front Sports Act Living. 2025 Feb 12;7:1505301. doi: 10.3389/fspor.2025.1505301 (PMC11861109; doi:10.3389/fspor.2025.1505301)
Supplement: Supplementary file 1 [file Table1.docx]

| **Table 1.** Main characteristics of included studies. | | | | | | | | |
| --- | --- | --- | --- | --- | --- | --- | --- | --- |
| **Author(s)** | **Country** | **Population** | **Type of intervention** | **Study design** | **Presence of comparison group** | **To-be-learned skill** | **Measurement instrument** | **Intervention duration** |
| **Traditional: use of video for instruction or feedback** | | | | | | | | |
| Bunker et al. (1976) | United States | **Number**  *n* = 36  **Age**  4.5-8.5 yrs. | Video feedback | Pretest-intervention-posttest | Yes (video + auditory feedback vs. auditory feedback) | Freestyle flutter kick | Perceived progress observed by expert | **Number of sessions**  *n* = 4  **Duration of one session**  *t* = 15 min  **Total duration**  *t* = 4 weeks |
| Clark & Ste-Marie (2007) | Canada | **Number**  *n* = 33  (*F* = 20, *M* = 13)  **Age**  8.3 (± 1.2) yrs. | Self-as-a-model intervention | Pretest-intervention-retention (24h) | Yes (self-modeling vs. video feedback vs. control) | 1. Stroke of choice (front crawl, back crawl, breaststroke, elementary backstroke, butterfly)  2. Motivational beliefs | 1. Perceived progress observed by expert  2. Questionnaires | **Number of sessions**  *n* = 6  **Duration of one session**  *t* = 30 min  **Total duration**  *t* = 6 days |
| Da Silva Pinto Marques-Dahi et al. (2016) | Brazil | **Number**  *n* = 20  (*F* = 12, *M* = 8)  **Age**  12 (± 0.63) yrs. | Video + verbal instruction | Pretest-intervention-retention  (1 week)-transfer | Yes (video + arm instruction vs. video + interaction arm and breathing instruction vs. video only) | Front crawl | Perceived progress observed by expert + kinematic measurements | **Number of sessions**  *n* = 4  **Total duration**  *t* = 2 weeks |
| Weiss et al. (1998) | United States | **Number**  *n* = 24  (*F* = 6, *M* = 18)  **Age**  6.2 (± 0.90) yrs. | Video instruction | Pretest-intervention-posttest-retention  (4 days) | Yes (peer-mastery vs. peer-coping vs. control) | 1. Aquatic skills (blowing bubbles, submersion, supported prone float, prone float with kick, front crawl, back float)   1. 2. Self-efficacy   3. Anxiety management | 1. Perceived progress observed by expert  2. Questionnaires  3. Questionnaires | **Number of sessions**  *n* = 3  **Duration of one session**  *t* = 20 min  **Total duration**  *t* = 3 days |
| **Contemporary: non-linear swimming program** | | | | | | | | |
| Invernizzi et al. (2021) | Italy | **Number**  *n* = 100  (*F* = 53, *M* = 47)  **Age**  5.9 (± 0.3) yrs. | Non-linear pedagogy | Pretest-intervention-posttest | Yes (linear pedagogy vs. non-linear pedagogy) | 1. Aquatic skills (Langendorfer & Bruya, 1995)  2. Perceived aquatic skills | 1. Perceived progress observed by expert  2. Questionnaires | **Number of sessions**  *n* = 30  **Duration of one session**  *t* = 50 min  **Total duration**  *t* = 15 weeks |
| **Table 1.** Continued. | | | | | | | | |
| **Author(s)** | **Country** | **Population** | **Type of intervention** | **Study design** | **Presence of comparison group** | **To-be-learned skill** | **Measurement instrument** | **Intervention duration** |
| **Atheoretical: learn-to-swim programs** | | | |  |  |  |  |  |
| Bitang et al. (2020) | Romania | **Number**  *n* = 16  (*F* = 4, *M* = 12)  **Age**  5-7 yrs. | Not specified | Pretest-intervention-posttest | No | Aquatic skills (floating, gliding on water surface, breathing, front crawl) | Perceived progress observed by expert | **Number of sessions**  *n* = 36  **Total duration**  *t* = 3 months |
| Calverley et al. (2022) | Australia | **Number**  *n* = 105  (*F* = 44, *M* = 61)  **Age**  Group 1: 8.1 yrs. Group 2: 10.9 yrs. | Not specified | Pretest-intervention-posttest | No | 1. Water safety knowledge  2. Aquatic skills (floating, swimming, safe water entry and exit, rescue skills, survival swimming) | 1. Questionnaires  2. Perceived progress observed by expert | **Number of sessions**  *n* = 10  **Duration of one session**  *t* = 60 min  **Total duration**  *t* = 5 days or 10 weeks |
| Čižas & Milašius  (2017) | Lithuania | **Number**  *n* = 25  (*F* = 13, *M* = 12)  **Age**  6.91 (± 0.58) yrs. | Not specified | Pretest-intervention-posttest | No | Aquatic skills (breathing, floating, gliding on water surface, breaststroke, backstroke, jumping into water) | Perceived progress observed by expert + kinematic measurements | **Number of sessions**  *n* = 20  **Duration of one session**  *t* = 45 min  **Total duration**  *t* = 10 weeks |
| Frankl (1996) | United States | **Number**  *n* = 78  (*F* = 39, *M* = 39)  **Age**  7-10 yrs. | Not specified | Pretest-intervention-posttest | No | 1.Aquatic skills (American Red Cross, 1996)  2.Self-esteem  3.Attitudes toward swimming | 1. Perceived progress observed by expert  2. Questionnaires  3. Questionnaires | **Number of sessions**  *n* = 21  **Duration of one session**  *t* = 35 min  **Total duration**  *t* = 1 year |
| Jurak et al. (2001) | Slovenia | **Number**  *n* = 370  **Age**  8-9 yrs. | Content and duration swimming program | Pretest-intervention-posttest | Yes (experimental 1 vs. experimental 2 vs. control) | Breaststroke | Perceived progress observed by expert | **Number of sessions**  *n* = 10 or 15  **Duration of one session**  *t* = 60 or 90 min  **Total duration**  *t* = 10 weeks |
| Kotliarov (2020) | Russia | **Number**  *n* = 40  (*F* = 20, *M* = 20)  **Age**  7-8 yrs. | Ratio swimming front- and back crawl | Pretest-intervention-posttest | Yes (60:40 ratio vs. 50:50 ratio) | Front and back crawl | Perceived progress observed by expert + kinematic measurements | **Number of sessions**  *n* = 80  **Duration of one session**  *t* = 45 min  **Total duration**  *t* = 10 months |
| **Table 1.** Continued. | | |  |  |  |  |  |  |
| **Author(s)** | **Country** | **Population** | **Type of intervention** | **Study design** | **Presence of comparison group** | **To-be-learned skill** | **Measurement instrument** | **Intervention duration** |
| Kováčová et al. (2019) | Slovakia | **Number**  *n* = 60  **Age**  8.63 yrs. | Short intensive swimming course | Pretest-intervention-posttest | No | Aquatic skills (jumping into water, flutter kick with kickboard 25 m, diving, and catching puck, swimming (freestyle or backstroke)) | Perceived progress observed by expert | **Number of sessions**  *n* = 5  **Duration of one session**  *t* = 45 min  **Total duration**  *t* = 5 days |
| Mirvić & Rasidagić (2017) | Bosnia & Herzegovina | **Number**  *n* = 245  (*F* = 0, *M* = 245)  **Age**  8-10 yrs. | Not specified | Pretest-intervention-posttest | No | Aquatic skills (safe water entry, submersion, underwater breathing, prone float, back float, gliding on water surface, jumping into water, swimming) | Perceived progress observed by expert | **Number of sessions**  *n* = 12  **Duration of one session**  *t* = 90 min  **Total duration**  *t* = 12 days |
| Moncrieff et al. (1962) | United States | **Number**  *n* = 54  **Age**  5-11 yrs. | Frequency swimming lessons | Pretest-intervention-posttest | Yes (6 sessions in 2 weeks vs. 6 sessions in 3 weeks) | Aquatic skills (submersion, vertical float, face float, back float, swimming) | Perceived progress observed by expert | **Number of sessions**  *n* = 6  **Duration of one session**  *t* = 25 min  **Total duration**  *t* = 2 or 3 weeks |
| Moura et al. (2021) | Brazil | **Number**  *n* = 31  (*F* = 15, *M* = 16)  **Age**  8.0 (± 0.86) yrs. | Focus of swimming program | Pretest-intervention-posttest | Yes (aquatic skills acquisition vs. swimming skills acquisition) | Aquatic skills (Langendorfer & Bruya, 1995) | Perceived progress observed by expert | **Number of sessions**  *n* = 12  **Total duration**  *t* = 12 weeks |
| Sheyko & Pashchenko (2018) | Ukraine | **Number**  *n* = 30  **Age**  6-8 yrs. | Aquatic games | Intervention- posttest | Yes (use of games vs. no use of games) | Front and back crawl | Perceived progress observed by expert | **Number of sessions**  *n* = 36  **Duration of one session**  *t* = 60 min  **Total duration**  *t* = 12 weeks |
| Susnara et al. (2022) | United States | **Number**  *n* = 200  (*F* = 88, *M* = 112)  **Age**  4-14 yrs. | Not specified | Pretest-intervention-posttest | No | 1. Water safety knowledge  2. Aquatic skills (American Red Cross, 2014)  3. Value for swimming | 1. Questionnaires  2. Perceived progress observed by expert  3. Questionnaires | **Number of sessions**  *n* = 16  **Duration of one session**  *t* = 40-50 min  **Total duration**  *t* = 4 weeks |
| **Table 1.** Continued. | | |  |  |  |  |  |  |
| **Author(s)** | **Country** | **Population** | **Type of intervention** | **Study design** | **Presence of comparison group** | **To-be-learned skill** | **Measurement instrument** | **Intervention duration** |
| **Atheoretical: learning** **environment** | | | | | | |  |  |
| Button et al. (2020) | New Zealand | **Number**  *n* = 98  (*F* = 44, *M* = 54)  **Age**  9.0 (± 1.3) yrs. | Open-water swimming | Pretest-intervention-posttest-retention  (3 months) | No | 1. Water safety knowledge  2. Aquatic skills (safe water entry and exit, floating, submersion, obstacle course, rescue skills, swimming) | 1. Questionnaires  2. Perceived progress observed by expert | **Number of sessions**  *n* = 13  **Total duration**  *t* = 3 days |
| Costa et al. (2012) | Portugal | **Number**  *n* = 98  **Age**  4.4 (± 0.5) yrs. | Water depth | Intervention-posttest  (6 months)-retention  (12 and 18 months) | Yes (shallow vs. deep water) | Aquatic skills (Langendorfer & Bruya, 1995) | Perceived progress observed by expert | **Number of sessions**  *n* = 144  **Duration of one session**  *t* = 40 min  **Total duration**  *t* = 18 months |
| Rocha et al. (2018) | Portugal | **Number**  *n* = 21  **Age**  4.70 (± 0.51) yrs. | Water depth | Pretest-intervention-posttest | Yes (shallow vs. deep water) | Aquatic skills (Langendorfer & Bruya, 1995) | Perceived progress observed by expert | **Number of sessions**  *n* = 48  **Total duration**  *t* = 6 months |
| **Atheoretical: use of assistive devices** | | | | | |  |  |  |
| Misimi et al. (2022) | Slovenia | **Number**  *n* = 40  (*F* = 20, *M* = 20)  **Age**  10.5 (± 0.5) yrs. | Use of goggles and snorkel in children with fear of water | Pretest-intervention-posttest | Yes (goggles and snorkel vs. no goggles and snorkel) | Aquatic skills (Langendorfer & Bruya, 1995) | Perceived progress observed by expert | **Number of sessions**  *n* = 5  **Duration of one session**  *t* = 45 min  **Total duration**  *t* = 4 weeks |
| Misimi et al. (2023) | Slovenia | **Number**  *n* = 40  (*F* = 20, *M* = 20)  **Age**  10.5 (± 0.5) yrs. | Use of goggles and snorkel in children without fear of water | Pretest-intervention-posttest | Yes (goggles and snorkel vs. no goggles and snorkel) | Aquatic skills (Langendorfer & Bruya, 1995) | Perceived progress observed by expert | **Number of sessions**  *n* = 5  **Duration of one session**  *t* = 45 min  **Total duration**  *t* = 4 weeks |
| Scurati et al. (2006) | Italy | **Number**  *n* = 20  **Age**  8-9 yrs. | Use of flotation devices | Pretest-intervention-posttest | Yes (flotation devices vs. no flotation devices) | Front crawl | Perceived progress observed by expert + kinematic measurements | **Number of sessions**  *n* = 10  **Duration of one session**  *t* = 40 min |
|  |  |  |  |  |  |  |  |  |

| **Table 2.** Aims, theoretical frameworks, and key findings of the included studies. | | | |
| --- | --- | --- | --- |
| **Author(s)** | **Aim(s)** | **Theoretical Framework(s)** | **Key findings** |
| **Traditional: use of video for instruction or feedback** | | | |
| Bunker et al. (1976) | To examine the effects of video feedback on the flutter kick swimming skill in two age groups: 4.5-6.5 years and 6.5-8.5 years. | None, but in the discussion, reference is made to Piaget's (1964) theory | The effectiveness of acquiring skills with video feedback was superior only for the oldest group (4.5-6.5 years: *F* = 1.5, *p* > 0.05; 6.5-8.5 years: *F* = 4.65, p < 0.05) |
| Clark & Ste-Marie (2007) | To examine the impact of two self-as-a-model interventions, namely self-modeling and video feedback, on children’s self-regulation of learning and swimming performance. | - Zimmerman’s (1989, 2000) triadic analysis of self-regulatory functioning  - Bandura’s (1977, 1986, 1997) social cognitive theory of observational learning | The self-modeling intervention demonstrated superior swimming performance (*F*(2,30) = 9.38, *p* = 0.001, eta_p_^2^ = 0.44), greater self-satisfaction (*F*(2,30) = 8.91, *p* = 0.001, eta_p_^2^ = 0.44), and higher intrinsic motivation (*F*(2,30) = 14.7, *p* = 0.0001, eta_p_^2^ = 0.49). Although not statistically significant, they also displayed a tendency towards greater self-efficacy beliefs (*F*(2,30) = 3.80, *p* = 0.03, eta_p_^2^ = 0.18) compared to both the video feedback and control interventions. No significant differences were found between the video feedback and control interventions (all *p* > 0.05). |
| Da Silva Pinto Marques-Dahi et al. (2016) | To investigate whether verbal instructions emphasizing the interaction between arm stroke and breathing in the front crawl enhance learning gains when combined with video demonstrations. | None | Enhancing a video demonstration with verbal instruction improves children's learning of the front crawl more effectively than providing the video alone (*F*(2, 17)= 3.72, *p* < 0.05, eta_p_^2^ = 0.30). Furthermore, verbal instructions on the interaction between arm stroke patterns and breathing may lead to even better learning outcomes compared to instructions focusing solely on arm stroke patterns (*F*(2, 17)= 3.72, *p* < 0.05, eta_p_^2^ = 0.30). |
| Weiss et al. (1998) | To examine the effects of peer-coping and peer-mastery models on fearful children's motor performance and psychological responses in the context of swimming. | Bandura’s (1977, 1986, 1997) social cognitive theory of observational learning | Peer-coping (1) and peer-mastery (2) demonstrated better aquatic skill learning (ES_1_ = -0.28, ES_2_ = -0.44), increased self-efficacy (ES_1_ = -1.22, ES_2_ = -0.50), and reduced fear of swimming (ES_1_ = 1.14, ES_2_ = 1.21) compared to the control intervention. Peer-coping showed superior self-efficacy outcomes compared to peer-mastery (ES = -0.94). |
| **Contemporary: non-linear swimming program** | | | |
| Invernizzi et al. (2021) | To compare the effects of a teacher-centered approach (linear pedagogy) and a student-centered approach (non-linear pedagogy) on motor acquisition and children’s and parent’s perceptions of swimming. | Non-linear pedagogy (Chow, 2013) | Children showed a preference for the non-linear approach, finding it more engaging, whereas the linear program led to greater progress (*p* < 0.05, phi = 0.08) and was deemed more rewarding by parents. |
| **Atheoretical: learn-to-swim programs** | | | |
| Bitang et al. (2020) | To evaluate the effectiveness of swimming means in the acquisition of swimming skills among children aged 5–7 years. | None | Swimming performance improved significantly from the pre-test (*M* = 3.93) to the immediate test (*M* = 5.07) and further to the post-test (*M* = 6.91). |
| Calverley et al. (2022) | To determine the feasibility and effectiveness of a new child-focused lifesaving, swimming, and water safety program delivered in inland regional areas of Victoria: ‘Bush Nippers’. | None | The study showed an increase in water safety knowledge among participants under 9 years old (*t*(56) = 3.271, *p* = 0.002, *d* = 0.4), but no change was observed among those under 12 (*p* = 0.091). The assessment of aquatic skills solely relied on post-test results without tracking learning progress. |
| **Table 2.** Continued. | |  |  |
| **Author(s)** | **Aim(s)** | **Theoretical Framework(s)** | **Key findings** |
| Čižas & Milašius  (2017) | To develop a program for the acquisition of swimming skills in children aged 6–7 years and to evaluate its effectiveness. | None | The applied program demonstrated a positive effect on children's acquisition of primary swimming skills. |
| Frankl (1996) | To evaluate the effectiveness of a program that teaches water safety principles, as well as swimming and diving skills, to children from low-income families on swimming and diving skills and self-esteem. | None | Swimming ability showed a significant improvement from the pre-test (*M* = 4.26) to the post-test (*M* = 6.06) (*t*(69) = 15.49, *p* < 0.0001). Self-esteem also increased significantly from the pre-test to the post-test (*t*(74) = 2.11, *p* = 0.038). Additionally, students' attitudes toward swimming improved significantly from the pre-test to the post-test (*t* = 3.7, *p* = 0.001). |
| Jurak et al. (2001) | To assess the effectiveness of two experimental instruction programs (with identical content but different durations) compared to a standard instruction program (featuring different content) in improving swimming skills. | None (apart from some pedagogical notions) | The experimental swimming instruction programs were superior to the control program (*p* = 0.32 between experimental 1 and control; *p* = 0.007 between experimental 2 and control) without being significantly different from each other (*p* = 0.587). |
| Kotliarov (2020) | To determine the optimal ratio of front crawl to back crawl instruction (e.g., 50/50% vs. 60/40%) for acquiring swimming skills in children aged 7-8 years. | None | The 60/40% ratio of front crawl to back crawl instruction was more effective for acquiring swimming skills than the 50/50% ratio (*t* = 3.2, *p* < 0.05). |
| Kováčová et al. (2019) | To examine the effect of a 5-day-long intensive swimming course on swimming acquisition for pupils from elementary school. | None | The five-day swimming course proved effective, with pupils showing significant improvement across all five basic swimming tests (all *p* < 0.0001). |
| Mirvić & Rasidagić (2017) | To evaluate the impact of a 24-hour program designed to let primary school children learn swimming skills. | None | Swimming performance improved significantly from the pre-test to the post-test (*t*(244) = -37.83, *p* < 0.0001). |
| Moncrieff et al. (1962) | To compare the effectiveness of six practice sessions distributed over three days per week versus two days per week on the acquisition of elementary swimming skills in novice swimmers. | None | No statistically significant differences were observed between the group practicing six sessions distributed over three days per week and the group practicing six sessions over two days per week in achieving the six elementary water skills (*p* > 0.05). |
| Moura et al. (2021) | To compare the effects of two learn-to-swim programs—one emphasizing aquatic skills acquisition and the other focusing on swimming skills acquisition —on aquatic readiness and motor coordination in Brazilian school-aged children. | None | Both swimming programs led to improvements in aquatic readiness and motor coordination, with greater improvements in aquatic competence observed after lessons that focused on aquatic skills (*F* = 24.19, *p* < 0.01, eta_p_^2^  = 0.46). |
| Sheyko & Pashchenko (2018) | To establish the effectiveness of using games in water to let primary school-aged children learn swimming skills. | None | The incorporation of aquatic games during swimming lessons resulted in improved front crawl and back crawl performance compared to lessons that did not utilize aquatic games. |
| Susnara et al. (2022) | To examine the impact of an out-of-school swimming program on children and youth from one underserved community. | Theories based on socialization research | The out-of-school swimming program led to significant enhancements in aquatic skills and water safety knowledge (*F*(1) = 130.71, *p* < 0.0001). Participants also developed a positive perception of swimming due to socialization, particularly influenced by the instructors. |
| **Table 2.** Continued. | |  |  |
| **Author(s)** | **Aim(s)** | **Theoretical Framework(s)** | **Key findings** |
| **Atheoretical: learning** **environment** | | | |
| Button et al. (2020) | To determine if the developed swimming program is effective for children to acquire and learn aquatic knowledge and skills in open-water environments. | None | Significant improvements were observed in the number of competent children, typically occurring from pre- to post-test and/or from pre-test to retention (all *p* < 0.017) |
| Costa et al. (2012) | To compare the effectiveness of shallow- versus deep-water swimming lessons for 4-5-year-old children in learning aquatic skills over a period of 6, 12, and 18 months of practice. | None | After 6 months of practice, shallow-water lessons resulted in greater water competence compared to deep-water lessons (Λ = 0.131, 𝜒2 = 43.778, *p* < 0.001). However, this difference was no longer evident after 12 (Λ = 0.395, 𝜒2 = 19.945, *p* = 0.277) and 18 months (Λ = 0.488, 𝜒2 = 17.240, *p* = 0.370) of practice. |
| Rocha et al. (2018) | To determine the effects of shallow- versus deep-water swimming lessons on the acquisition of preschoolers’ aquatic skills after 6 months of practice | None, but constructivism (Piaget, 1964) is mentioned | Shallow-water swimming lessons led to greater aquatic competence in preschool children after 6 months of practice (Λ = 0.119, 𝜒2 = 36.124, *p* < 0.001). |
| **Atheoretical: use of assistive devices** | | | |
| Misimi et al. (2022) | To examine the effects of using goggles and snorkel during swimming lessons on the aquatic skills of young non-swimmers with fear of water | None, but the constraints-led approach is mentioned (Davids et al., 2008) | The use of goggles and a snorkel appears to be more beneficial in swimming lessons for young swimmers with a fear of water compared to lessons that do not involve using goggles (*p* < 0.05), except for activities like blowing bubbles (*F*(0.83, 0.56) = 4.39, *p* = 0.04). |
| Misimi et al. (2023) | To examine the effects of using goggles and snorkel during swimming lessons on the aquatic skills of young non-swimmers without fear of water | None, but the constraints-led approach is mentioned (Davids et al., 2008) | The use of goggles and snorkel had no significant effect on most aquatic skills of young non-swimmers without a fear of water (*p* > 0.05). |
| Scurati et al. (2006) | To examine the effectiveness of instructional flotation devices on the acquisition of front crawl swimming skills in Italian children aged 8-9 years. | None | The acquisition of the front crawl is not significantly influenced by the use or non-use of instructional flotation devices (*p* > 0.05). |
